# Eye movement as a simple, cost-effective tool for people who stutter: A case study

**DOI:** 10.4102/sajcd.v70i1.968

**Published:** 2023-08-31

**Authors:** Hilary D.-L. McDonagh, Patrick Broderick, Kenneth Monaghan

**Affiliations:** 1 Department of Health and Nutritional Science, Faculty of Science, Atlantic Technological University, Sligo, Ireland; 2 Department of Social Science, Faculty of Business and Social Science, Atlantic Technological University, Sligo, Ireland

**Keywords:** stuttering, intervention, self-help, telepractice, eye movement, saccades, tongue, anticipation, holistic, accessible, affordable

## Abstract

**Background:**

Access to services remains the biggest barrier to helping the most vulnerable in the South African Stuttering Community. This novel stuttering therapy, harnessing an unconscious link between eye and tongue movement, may provide a new therapeutic approach, easily communicated and deliverable online.

**Objectives:**

This study provides both objective and subjective assessments of the feasibility of this intervention. Assessment tools holistically address all components of stuttering in line with comprehensive treatment approaches: core behaviours, secondary behaviours, anticipation and reactions.

**Method:**

On receipt of ethical approval, this single-subject case design recruited one adult (21-year-old) male with a developmental stutter (DS). The participant gave informed consent and completed four scheduled assessments: baseline, after 5-week training, 3 months post-intervention and 24 months post-completion. The study used objective assessment tools: Stuttering Severity Instrument-4 (SSI-4); Subjective-assessment tools: SSI-4 clinical use self-report tool (CUSR); Overall Assessment of Speaker’s Experience of Stuttering (OASES-A); Premonitory Awareness in Stuttering (PAiS) and Self-Report Stuttering Severity* (SRSS) (*final assessment).

**Results:**

The participant’s scores improved across all assessment measures, which may reflect a holistic improvement. The participant reported that the tool was very useful. There were no negative consequences.

**Conclusion:**

This case report indicates that this innovative treatment may be feasible. No adverse effects were experienced, and the treatment only benefited the participant. The results justify the design of a pilot randomised feasibility clinical trial.

**Contribution:**

The results indicate that this is a needed breakthrough in stuttering therapy as the instructions can be easily translated into any language. It can also be delivered remotely reducing accessibility barriers.

## Introduction

This descriptive case report explores whether making a specific, timed, downward eye movement is effective as a holistic intervention for a young adult man with a developmental stutter (DS). This novel self-help intervention aims to enable the person who stutters to circumvent an inadvertent block or repetition, should they choose to do so. It harnesses an unconscious synchronised linking of eye movement and the posterior of the tongue, discussed by the main authors in a previously published paper (McDonagh & Monaghan, 2019). Accessibility to speech-language pathology across South Africa has always been a key barrier to helping the most vulnerable (Khoza-Shangase & Mophosho, 2018), and there has been a call for research evidence that can help facilitate a culturally, linguistically and contextually compatible service delivery, within the population and context (Khoza-Shangase & Mophosho, 2021). Current approaches to treating stuttering (Brignell et al., 2020; Connery et al., 2021) can be costly for individuals and overload speech pathology services (Meredith et al., 2023). Specifically, speech restructuring programmes such as the Comprehensive Stuttering Programme (Karani & Mupawose, 2020; Langevin et al., 2010; Scott Yaruss et al., 2012a) and the Camperdown Programme (Carey et al., 2010, 2012), which have the most empirical evidence, are difficult to maintain post-treatment. Participants frequently relapse to their original speaking patterns, which results in adults who stutter repeatedly accessing services in the form of maintenance (Blumgart et al., 2010). By expanding on this new line of research, the opportunity may exist for researchers and professionals in the speech, language and hearing professions to provide an intervention applicable to all within South Africa’s diverse stuttering population (Khoza-Shangase & Mophosho, 2018).

A DS, which typically starts as language skills develop between 3 and 5 years of age, affects an estimated 5% – 8% of children worldwide (Reilly et al., 2009). Disrupted speech, the core stuttering behaviour, is characterised by involuntary word or part-word repetitions, sound prolongations and silent blocks that actively disrupt the production of overt speech and impair communication (World Health Organization [WHO], 2016). In most cases, DS does not persist past childhood, but a lifetime prevalence has been estimated at almost 1% (Craig et al., 2002; Yairi & Ambrose, 2013). Holistically, the negative experience of a DS extends far beyond disrupted speech. For example, people who stutter (PWS) experience a range of abnormal physical movements known as concomitant or secondary behaviours. These include upper limb and body movements, facial grimaces, involuntary eye movements, blinks and shortness of breath (Didirkova et al., 2019; Kosmala et al., 2019). However, these secondary behaviours vary considerably across individuals with a study of 85 PWS from Argentina identifying 66 different abnormal movements (Riva-Posse et al., 2008).

By adulthood, most stuttering-like moments can be predicted, and a variety of linked coping responses automated: some constructive and some not. An American study of 30 adults with DS identified both action (cue to employ a speech technique or avoidance behaviour) and non-action (cognitive, emotional and affective) responses in the anticipation of stuttering (Jackson et al., 2015), with 43% of the participants reporting that anticipation produced both positive and negative results, whereas 37% reported that anticipation was not at all helpful. Specifically following the anticipation of a stuttering moment, 87% of participants avoided sounds or words, 37% avoided situations and 23% chose not to speak in preference to stuttering.

Internationally, it has been proposed that stuttering treatment should focus on facilitating productive responses to anticipation of a stuttering event (Briley, 2016; Tichenor et al., 2022). Current clinical approaches do this by addressing the three components of stuttering: overt core behaviours, secondary behaviours, and negative feelings and attitudes (Guitar, 2014). Such interventions focus on individualised (Sønsterud et al., 2020) and comprehensive interventions (Karani & Mupawose, 2020; Yaruss et al., 2012). Other clinicians advocate focusing on stuttering acceptance and communication competence (Byrd et al., 2022). As all these approaches include a focus on cognitive, emotional and affective personal reactions, they are, by linguistic design, more suitable to those accessing treatment through the private sector in South Africa.

A South African qualitative study (Barratt et al., 2012) explored whether the linguistic complexity of an assessment tool, designed in English, was equitably translated by five different local translators translating from English to isiZulu. Their findings revealed differences relating to translation of both vocabulary and semantics, key ingredients in any affective intervention, leaving speech and language professionals struggling to equitably provide services. Therefore, in the public sector, treatment provision to those who have accessed services has been challenged by the linguistic diversity of the nation. Furthermore, a 2022 study (Tar-Mahomed & Kater, 2022) highlighted that within the South African context, the geographical challenges of technological access and supportive infrastructure are not the same across the healthcare sector. These authors reported that the private healthcare sector is better resourced than the public sector.

There is a need for a new approach, and this requires a new conceptualisation of what occurs at stuttering-like moments. At a neurological level, all behaviours are first learned, then predicted and then implemented (Tenison et al., 2016). Even though we are not consciously aware of it, our eyes are constantly moving (Diaz et al., 2012). These movements are called saccades and are directly linked with our learned behaviour patterns and implemented by our unconscious. As our unconscious considers options and prepares us for action, our eyes identify the targets and required vectors of possible actions with lightning-fast movements that last between 20 milliseconds (ms) and 200 ms. Patterns of saccadic eye movement are as unique to individuals as their fingerprints (Lohr & Komogortsev, 2022). As our eyes move first, and eye and tongue movements are synchronised, this new conceptualisation contends that the most effective approach to change from an inhibited unconsciously generated movement may be a conscious eye movement. Unlike other treatment approaches, this treatment addresses only eye movement, identified as the start of all predicted actions.

If a conscious eye movement enables speech motor control, this tool could empower PWS to choose not to stutter and still speak naturally. It is not a tool to simply modify a stuttering moment. Rather, it should prevent it as, by changing the prediction, the primary symptom of stuttering should not occur. If a PWS is given the choice to voluntarily use this tool and not stutter, regardless of environment, personal reactions and the negative impact typically aligned with involuntary stuttering moments should by themselves diminish. Stuttering, also known as a stammer in India, United Kingdom and Ireland, is characterised by involuntary repetition or prolongation of sounds, syllables or words, or by voluntary hesitation or pauses, that disrupt the smooth rhythmic flow of speech (WHO, 2001), and it is classified as a disability (WHO, 2016) when its severity is such as to markedly disturb the fluency of speech. For the person who stutters, however, the experience of stuttering extends far beyond these observable dysfluencies. To address this, the International Classification of Functioning, Disability and Health (ICF) (World Health Organization [WHO], 2001) was specifically adapted to apply to stuttering (Yaruss & Quesal, 2004) and adopted as a framework for SLP practice (American Speech-Language-Hearing Association [ASHA], 2016). The experience of stuttering has been conceptualised in terms of component parts: primary symptoms, personal reactions and adverse impact, all under the influence of environmental factors (see Tichenor et al., 2022, for schematic). The primary symptom identified in this schematic is the perceived loss of speech motor control under the influence of stuttering etiology. The ICF provides additional guidelines addressing participation restriction, activity limitation and locus of control. These constructs are self-assessed by the participant throughout the study.

This is a descriptive, single-subject case study design and provides an initial platform to explore the effectiveness of this new treatment approach for the participant. Assessments are of the same individual at different time points. The core dependent variable in this study is the experimental effect making a controlled eye movement has on stuttering severity. Assessments took place at four time periods: baseline, following the 5-week intervention, 3 months post-intervention and 24 months post-intervention. As such, the data presented reflect and evaluate the generalisability of the technique, by the participant, in real life post-intervention. While a descriptive case study represents a low level of evidence (Murad et al., 2016), it is the essential starting point for a new approach, to assess its feasibility and ensure no adverse effects. Comparable evidence exists from a recently published descriptive case study describing a comprehensive stuttering intervention for DS (Karani & Mupawose, 2020).

Assessment measures used to document the value of this intervention have considered the holistic nature of the disorder and the participant’s ability to use it in real life. Core and secondary behaviours were independently assessed online because of the coronavirus disease 2019 (COVID-19) lockdown. Stuttering severity was also self-assessed by the participant. Additionally, the participant was asked to assess their anticipation of stuttering events, the overall experience of being someone who stuttered and any changes in the frequency of avoidance behaviours and perceived locus of control. The data presented reflect the participant’s ability to use the technique in real life over the 2 years following the 5-week instruction period.

The approach used in this study is to familiarise the participant with the process of using a conscious eye movement to change their posterior tongue position before commencing utterance of the next speech sound. During training, it is just this movement that is practised and the timing of it discussed. The required positioning in the back of the tongue occurs when they move their eyes downwards towards their naval. This ‘ideal’ tongue position is with the posterior of the tongue raised and as far back in the oral cavity as possible. Having the tongue positioned on the roof of the mouth has been shown to improve muscle strength (Di Vico et al., 2013) and heart rate variability (Schmidt et al., 2009). It is the tongue position that naturally coincides with putting a big silly grin on your face.

Like improving stance for a better golf swing or a pre-kick routine for taking a penalty, the purpose of the practice is to make this starting position easy to achieve under pressure. The participant is advised not to use the tool outside of practice during the 5-week training period. After the training, the participant is free to keep up the practice if they find the tool useful. Five weeks was deemed long enough time for the novelty of the movement not to be a factor when attempting to use it in stressful situations, but short enough that the technique could be easily unlearned if the participant did not find it beneficial. This was a necessary precaution given the novelty of the approach.

This novel approach may present several opportunities to effectively treat the more vulnerable in South African society. Firstly, as this is a self-help intervention, it can be delivered online cost-effectively, highlighting the outreach possibilities of using it with PWS who do not have access to the mainstream, comprehensive approach and in line with the first recently identified core component of stuttering interventions (Connery et al., 2022). It has been stated that despite the implementation difficulties associated with telepractice in South Africa, the benefits far outweigh the challenges encountered (Karrim et al., 2022). Secondly, with treatment provision to those who have accessed services challenged by the linguistic diversity within South Africa (Barratt et al., 2012), this intervention is based on instructions for physical movement, which are easily translated. Thirdly, the online nature of the training and assessments used in this research may allow researchers in Speech and Language to reimagine research in a way that is applicable to the South African Population and Context (Khoza-Shangase & Mophosho, 2021). With further replications, the evidence base for this new treatment approach would be strengthened so that this novel intervention could be a tool for Speech and Language professionals in South Africa to improve the quality of life for the stuttering community. If uncertainty and perceived lack of control are predictors of communicative participation and mental health in adults who stutter (Boyle & Chagachbanian, 2022), a tool that empowers a PWS with the conscious choice to speak freely should address stuttering in a holistic manner.

## Methods

### Objectives

- To describe the scores obtained on the Stuttering Severity Instrument – Fourth Edition (SSI-4) for core and secondary behaviours (Phases 1, 3, 4 and 5).
- To describe the scores obtained on the Overall Assessment of the Speaker’s Experience of Stuttering-Adults (OASES-A) regarding its influence on activity limitations, participation restrictions and contextual factors (personal and environmental) (Phases 1, 3, 4 and 5).
- To describe any changes to the self-reported stuttering severity, avoidance behaviours and locus of control measures assessed through the SSI-4 auxiliary Clinical Use Self-Report (CUSR). These measures reflect the ICF guidelines regarding participation, avoidance behaviours and perceived locus of control (Phases 1, 3, 4 and 5).
- To describe the scores obtained on the Premonitory Awareness in Stuttering (PAiS) with regard to immediate and prospective anticipation (Phases 1, 3 4 and 5).
- To compare results provided on self-rated stuttering severity (SRSS) with SSI-4 scores. This measure was included only at 24 months post-intervention assessment (Phase 5).

### Research design

**FIGURE 1:**
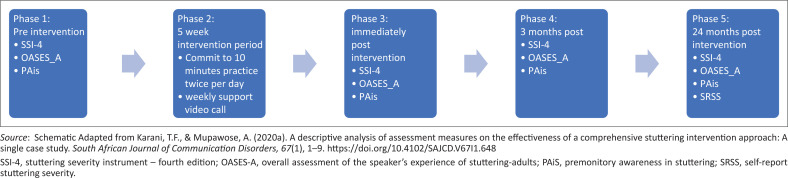
Five phases of the study procedure.

This study used a single-subject case design including assessments at four time periods (pre-intervention, immediate post-intervention, 3 months post-intervention and 24 months post-intervention) and a 5-week intervention period (see Figure 1).

#### Participant description

The participant was a right-handed male Caucasian, age 21 years, who was an undergraduate student at the Institute of Technology in Sligo, Ireland. He does not know at what age he started to stutter, just that he has stuttered for as long as he can remember. He had participated in some classes for speech in childhood without much success, and at age 19, he took part in an intensive 3-day training with the Maguire programme: a self-help programme run by PWS. He still uses the techniques he learned from the programme but finds them hard to remember. He was proficient in English and had no diagnosis, needing the psychological or medical intervention of any emotional, behavioural, learning or neurological disorder. He had the cognitive ability to take part and gave informed consent.

#### Data collection tools

The tools used at the four testing periods (Phase 1, Phase 3, Phase 4 and Phase 5) were SSI-4, OASES-A and PAiS. The SRSS was added at 24 months follow-up (Phase 5).

The SSI-4 is the most widely used syllable-based procedure for assessing overt stuttering symptoms (Riley, 1994, 2009). The SSI-4 overall score combines the following three sections: firstly, scaled percentage of stuttered syllables (%SS); secondly, the average duration of the three longest stuttering moments, timed to nearest one-tenth of a second and converted to a scale 2–18; and finally, the physical concomitants, secondary symptoms: distracting sounds, facial grimaces, head movements and movements of the extremities converted to scale scores of 0–20, observed at the time of symptom assessment. This combination gives an overall scaled stuttering severity score and percentile ranking (Riley, 1994, 2009).

The minimum detectable change (MDC) in the percent of stuttered syllables (%SS) has been set as 1% (Jones et al., 2005), as this would be the smallest change noticeable by the participant. It has been proposed that to be clinically significant, an intervention should result in a 50% reduction in stuttering (Reddy et al., 2010), although some researchers have considered 20% (Maguire et al., 2010) clinically significant. All evaluations using SSI-4 were carried out on two consecutive days at each assessment phase, and the average of these two scores was included in this report. Assessments were carried out via Zoom by an independent psychologist available and qualified to complete the assessments during the lockdown at the start of the COVID-19 pandemic. Community Speech and Language Therapists had, at that time, been redeployed within the health sector. The same assessor was used at all phases for consistency. The total score is a scaled measure that includes %SS, duration timed to the nearest one-tenth of a second and converted to a scale score of 2–19.

The SSI-4 includes an auxiliary self-report assessing 13 items on a 9-point scale. The 13 items when subdivided yield three categories: self-assessed stuttering severity, locus of control and frequency of avoidance behaviours (Tahmasebi et al., 2018) to reflect the ICF framework. There is no established minimum clinically important difference (MCID) or MDC.

The OASES-A (Yaruss & Quesal, 2004) is a validated and reliable questionnaire designed to document improvement and evaluate treatment efficacy and is based on the WHO’s International Classification of Functioning, Disability, and Health (ICF) (World Health Organisation, 2016). The MCID and MDC have not yet been estimated. The OASES scores have been reported as remaining relatively stable over time (Constantino et al., 2020) unlike severity ratings.

The PAiS is a 12-item questionnaire assessing immediate and prospective anticipation of stuttering (Cholin et al., 2016) that was adapted from the reliable and validated Premonitory Urge for Tics Scale (PUTS) (Woods et al., 2005). Changes in PAiS are described in terms of total scores and immediate and prospective anticipation scores.

The self-rated stuttering severity (SRSS) required participants to score their typical SRSS for eight individual representative speaking situations, on a scale of 1–9 (where 1 = no stuttering and 9 = extremely severe stuttering) that represents typical, best and worst stuttering severity in that situation. The mean of the eight scores was used in the analysis. This tool was added to the assessment at phase 5 to assess whether an expert in stuttering’s self-assessment, concurred with the final independent assessment of stuttering severity. Minimum clinically important difference and MDC can be approximated based on established measures for SSI-4 using the comparison table published by Jones et al. (2004).

#### Study procedure

**Phase 2: Eye movement training for stuttering intervention:** The intervention was delivered via Zoom, at the start of the COVID-19 pandemic. The intervention took place over a 5-week period. A practice Zoom session was conducted prior to commencing the intervention schedule. Total contact time for participant and researcher over five weeks training, was 1 h 17 min (excluding time spent on assessments).

During meeting one, the participant was trained on how to use eye/tongue movement to produce non-stuttered speech. The participant was instructed to practise this movement when he experienced dysfluencies for 10 min twice per day for the next week. As a result of the lockdown, the participant did not experience stuttered speech at home alone or with family during the first week. Based on this feedback, the practice schedule was adapted in the second week, initially to an online practice schedule with the principal researcher and then back to self-directed, as it became apparent that the movement could be practised in the absence of stuttered speech.

The remaining online sessions were support sessions where the participant confirmed that he had complied with the practice schedule and the precise timing of the movement was discussed: if a stuttering-like moment started, stop talking, reposition the tongue and start again.

Key instructions from training sessions:

- Attention was brought to linked eye–tongue movement – non-vision related as occurs with eyes closed.
- Role of eye movement in unconscious predicting movement was discussed.
- Ideal tongue position was discussed – practice to reposition quickly – like a blink.
- Consider the timing of movement – before speech gesture.
- The participant was reminded that this was a tool he could use if he wished to avoid a stuttering-like moment and that it would get easier with practice. It was not something he had to do, but something he could do if it helped.
- First-week instructions were to practise with speech, second and subsequent weeks – no speech was required to practise eye–tongue movement.

### Data analysis

Responses from SSI-4 and OASES-A were analysed based on total scores, impact scores and severity ratings using descriptive statistics. The CUSR adjunct to the SSI-4 was analysed by creating composite scores assessing self-rated stuttering severity, avoidance behaviours and locus of control (Tahmasebi et al., 2018).

Responses from the Premonitory Awareness in Stuttering were presented based on the total score of 12 validated questions. The subtotal scores for questions 1–6, commencing with ‘Right before I stutter’, and for questions 7–12 are also presented separately.

For the above assessment tools, % change from baseline is calculated for phases 3–5 using the formula:


([baseline score{Phase 1}]−[score at assessment phase{3,4 or 5}]/[baseline score])×100 [Eqn 1]

The self-rating stuttering severity assessment tool was introduced in Phase 5 and its scores were compared to the SSI-4 based on the comparative table in O’Brian et al.’s (2004) scores to assess the internal validity of the measures and the reliability of the applicability of the SRSS in this and future studies. This also includes satisfaction with speech and frequency of avoidance behaviours measures.

### Ethical considerations

Ethical clearance to conduct this study was obtained from the Institute Research Ethics Committee (IREC), the Institute of Technology Sligo (now Atlantic Technological University Sligo) and the Sligo University Hospital Research Ethics Committee (reference no.: 800).

## Results

The results are presented to reflect the five study objectives described in the ‘Methods’ section.

### Objective 1

To describe the SSI-4 scores for core and secondary behaviours (see Table 1).

**TABLE 1:** Categorical description of measurements used in the Stuttering Severity Instrument – Fourth Edition Assessment.

Score	Description of measure
%SS	Raw % score reading + raw % score speaking combined
SSI-4 scaled severity score	SSI-4 score overall % stuttered syllables converted to a scale of 2–18
Frequency/duration longest	Timed to the nearest 1/10th of a second and converted to a scale score of 2–18
Physical concomitants	Distracting sounds, facial grimaces, head movements and movements of the extremities are scored on a scale of 0–20
**SSI-4 score**	
**Total of scaled scores**	
Naturalness of speech	Likert scale of 1–9, 9 being the least natural and 1 being the most natural
SSI-4 category	Verbal categorisation

%SS, percentage of stuttered syllables; SSI-4, Stuttering Severity Instrument – fourth edition.

**FIGURE 2:**
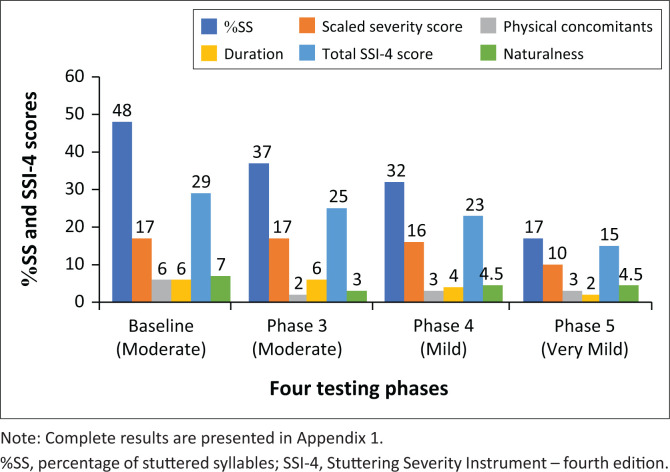
Stuttering Severity Instrument – 4 raw %SS, scaled section scores and total scores at four testing phases (see Table 2 for summary scores).

The SSI-4 scores (Figure 2 and Table 2) at the four testing phases are presented. The raw score of %SS decreased consistently across the four testing periods with the Phase 5 score representing a 65% change from baseline. This continuous reduction is not evident between Phase 1 and Phase 3 in the SSI-4 scaled score for %SS, which did not change (17 for both) even though there was an 11% reduction in %SS. Overall, the total SSI-4 score, and categorisation, improved from moderate (48) to very mild (17) at the final assessment. All component scores improved. Physical concomitants score (on a scale of 2–20) had decreased by 50% at Phases 4 and 5 (from 6 to 3). The scaled severity of the longest dysfluency (scale 2–18) had reduced from six to two at Phase 5 (70% change). The perceived naturalness of speech also improved changing from 7 (Phase 1) to 4.5 (Phase 5) (for full assessment, see Appendix 1).

**TABLE 2:** Stuttering Severity Instrument – Fourth Edition: Raw and scaled scores across all testing phases.

Description	Baseline Phase 1	Post-intervention Phase 3	3-month follow-up Phase 4	24-month follow-up Phase 5
%SS % of stuttered syllables	48	37	32	17
SSI-4 scaled stuttering severity	17	17	16	10
Scaled physical concomitants	6	2	3	3
Scaled frequency/duration	6	6	4	2
SSI-4 score / SSI-4 categorisation	29 Moderate	25 Moderate	23 Mild	15 Very mild
Naturalness of speech	7	3	4.5	4.5

%SS, percentage of stuttered syllables; SSI-4, Stuttering Severity Instrument – fourth edition.

### Objective 2

To describe the scores obtained on the Overall Assessment of the Speaker’s Experience of Stuttering – Adults (OASES-A) with regard to its influence on activity limitations, participation restrictions and contextual factors (personal and environmental).

**FIGURE 3:**
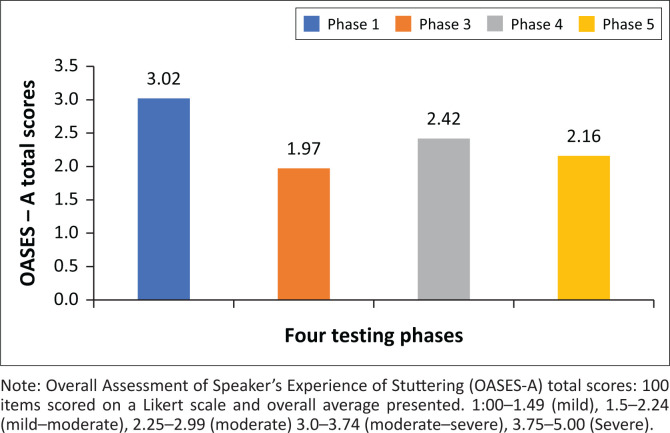
Overall Assessment of the Speaker’s Experience of Stuttering – Adults total impact scores.

The OASES-A scores and categorisation (Figure 3) improved from moderate–severe (3.1) at baseline to mild–moderate (2.2) at the final assessment. The improvement was not continuous as the initial gain (score 2 at Phase 3) was not fully maintained at Phase 4 (2.4); however, at Phase 5, the score (2.2) represented an overall improvement of 29% from baseline in total OASES-A score (see Appendix 2).

Section 1 (general information) did not improve overall, maintaining a moderate-severe negative impact (see Table 4). This section’s score showed initial improvements, a 30% reduction (Phase 3), decreased to a 25% reduction (Phase 4) and 2% by the final assessment (Phase 5). Specifically, initial improvements in general knowledge about stuttering reverted to baseline by 24 months follow-up (see Table 3).

**TABLE 3:** Overall Assessment of the Speaker’s Experience of Stuttering – Adults total impact scores at four assessment phases.

Study phase	Phase 1	Phase 3	Phase 4	Phase 5
Total/*n* = number answered	402/98	195/99	237/98	255/98
Impact score	3.02	1.97	2.42	2.16
Impact rating	Moderate–severe (3.0–3.74)	Mild–moderate (1.5-2-24)	Moderate (2.25–2.99)	Mild–moderate (1.5-2-24)
% reduction from the baseline section total score	-	35	20	29

**TABLE 4:** Overall assessment of the speaker’s experience of stuttering – Adults sectional results (% reduction in score from baseline).

Section	Baseline (Phase 1)	Post-intervention (Phase 3)		3-month follow-up (Phase 4)		24-month follow-up (Phase 5)	
		*n*	%	*n*	%	*n*	%
1. General Information	3.2	2.2	33	2.5	24	3.1	2
2. Speaker’s Reactions	3.4	2.1	39	2.8	19	2.8	19
3. Daily Communication	2.4	1.9	20	2.0	17	2.2	10
4. Quality of Life	3.1	1.9	40	2.5	21	2.0	37
5. Overall Impact	3.1	2.0	35	2.5	20	2.2	29
Overall Categorisation	Moderate–severe	Mild–moderate	-	Moderate	-	Mild–moderate	-

Note: Overall Assessment of Speaker’s Experience of Stuttering (OASES-A) with scales 1:00–1.49 (mild), 1.5–2.24 (mild–moderate), 2.25–2.99 (moderate), 3.0–3.74 (moderate–severe) and 3.75–500 (severe).

Section 2 (Personal Reactions) improved from moderate–severe (3.4) to mild–moderate (2.1) at Phase 3 and then stabilised at moderate (2.8) at phases 4 and 5 (see Table 4). The final % change from baseline was 19%. Of specific relevance, the participant reported that he strongly disagreed with the statement ‘when I stutter there is nothing, I can do about it’ at phases 3, 4 and 5 – this changed from a neutral reaction in Phase 1 (see Appendix 1).

Section 3 (Communications in Daily Situations) improved from moderate to mild–moderate at phases 3, 4 and 5. Within the range of this category, the initial % change from baseline was 20% (Phase 3), which decreased to 17% at Phase 4 and again to 10% at Phase 5.

Section 4 (Quality of Life Scores) mirrored the change in the overall OASES-A score, improving from moderate–severe to mild–moderate. Four of the 25 measures in this sub-section remained at returned to baseline by Phase 5; however, 21 of the 25 measures assessing the participant’s Quality of Life improved. This was not a continual improvement. The participant’s score had reduced by 40% from baseline at Phase 3, it was 21% at Phase 4, and it had 37% change from baseline at Phase 5 (see Table 4).

### Objective 3

To describe the SSI-4 CUSR measures. Composite scores of self-reported stuttering severity, avoidance behaviours and locus of control measures were assessed through the SSI-4 to reflect the ICF guidelines regarding participation and avoidance behaviours as well as the perceived locus of control.

**FIGURE 4:**
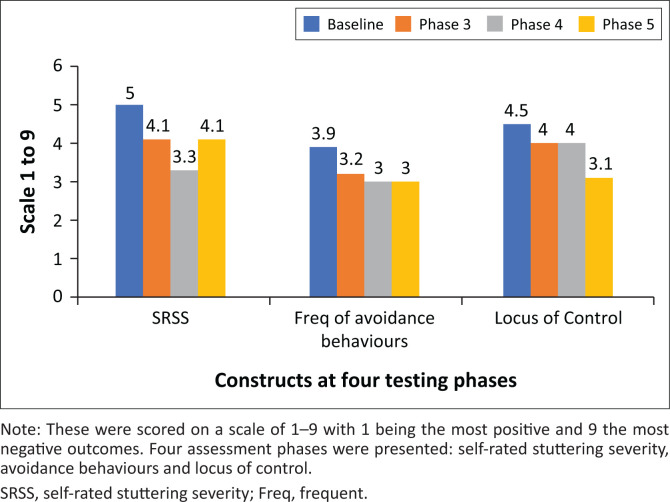
Details of composite scores from Stuttering Severity Instrument – Fourth Edition clinical use self-report tool.

The participant self-reported (Figure 4) that his stuttering severity had improved at Phase 3 (19% change from baseline) and that the improvement had been maintained at Phase 5 (19% change). At 3-month follow-up, perceived improvement peaked at 35%, but this level was not sustained. He also reported an initial reduction of 19% in his frequency of avoidance behaviours (Phase 3), and this reduction had further improved to 24% at phases 4 and 5. The participant reported an increased internalisation of the locus of control which improved continuously from the baseline assessment. Initial improvements were small, 10% and 11% at phases 3 and 4, but this continued to improve with a reported 31% increase in internal locus from baseline at Phase 5 (Full results are available from the corresponding author on request).

### Objective 4

To describe the scores obtained on the PAiS with regard to total scores and immediate or prospective anticipation scores. Coinciding with an international increase of interest in the role of anticipation in the stuttered speech, the PAiS (Cholin et al., 2016) was adapted from a validated and norm-referenced tool PUTS (Woods et al., 2005) exploring the premonitory sensations preceding Tics. The 12 questions deal with immediate and prospective awareness. When this tool was validated with PWS, there was an inverse relationship between the overall score and the percentage of stuttered syllables.

**FIGURE 5:**
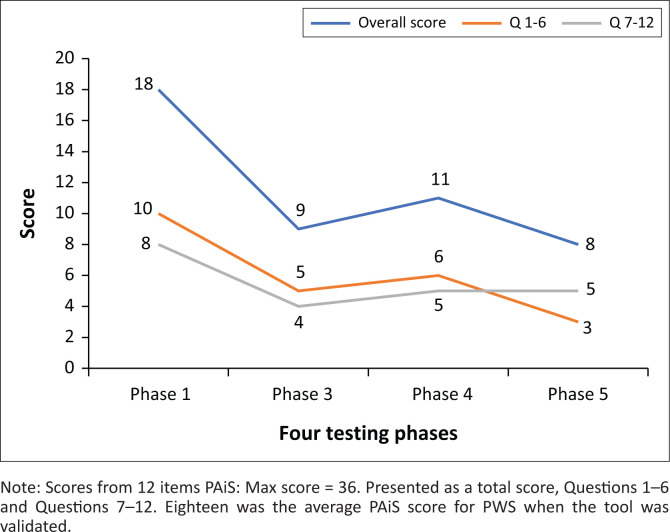
Premonitory awareness in stuttering.

As shown in Figure 5, the overall PAiS score reduced by 50% from baseline at Phase 3. Following a slight regression to 39% change from baseline at Phase 4, there was an overall 56% reduction from the baseline score at Phase 5 (Full results are available on request from the corresponding author).

Questions 1–6 are taken to reflect immediate anticipation of the stuttering event as they all commence with the words ‘right before I stutter’. The score on these questions decreased across the four testing periods, and the sub-score of immediate anticipation (Questions 1–6) was the largest contributor to the overall reduction in PAiS score having decreased 70% from baseline at Phase 5.

### Objective 5

To compare results provided on self-rated stuttering severity (SRSS) with SSI-4 scores. This measure was included only at 24 months post-intervention assessment (Phase 5).

The participant rated his satisfaction with speech at 2 on a scale of 1–9 with 1 = extremely satisfied and 9 = extremely dissatisfied. He rated their level of avoidance at 1.25 on a scale of 1–3 with 1 = rarely and 3 = most of the time. He also rated their typical worst and best severity on a scale of 1–9 with 1 fluent and 9 extremely severe stuttering. These scores are converted to comparative %SS based on published analysis (Jones et al., 2005) with a typical severity of SRSS 4.6 corresponding to approximately %SS = 8.6 and ranging from 3.4 (best) to 5.6 (worst) (Full results are available on request from the corresponding author).

Comparing these to the final %SS via SSI-4, the objective assessment of %SS via SSI-4 when speaking was greater (16.55%) than self-assessed severity (12.6% approximately) and when reading the actual performance was better (0.53%) than self-assessed best (5% approximately). The average of those scores is 8.54% corresponding well with the SRSS typical severity (8.6% approximately). This supports the use of typical SRSS when expanding the research.

## Discussion

The discussion elaborates on the objectives of the study and their respective findings.

### Objective 1

To describe the scores obtained on the SSI-4 for core and secondary behaviours.

The objective assessment of the participant’s Severity Ratings implied an improvement from moderate to very mild at the final assessment. It is important to recognise that only unconscious tongue position (linked with unconscious eye movements) was potentially modified or targeted in this intervention. While the intervention did not specifically deal with modifying speech in any way, objective assessment reported the participant reduced %SS, duration of longest dysfluency, physical concomitants and SSI-4 total scores (> 50% reduction from baseline). The %SS in reading task decreased by 97%. In the speaking task, the reduction was 46% at the final assessment. This difference is reflective of the inherent variability of stuttering severity across different contexts: online assessment, reading versus speaking tasks, social settings, etc., and would suggest that the eye movement enables fluent speech but is more difficult to use when trying to maintain eye movement for communication in social situations. %SS is presented rather than the scaled SSI-4 score because of its lack of sensitivity as a tool. Initial improvements in %SS were not reflected in a change in the SSI-4 verbal category because of the skewed nature of the population of PWS. Assessments between Phase 1 and Phase 3 show a 10.9% reduction in %SS, but the scaled SSI-4 score (17) would have remained the same (see Appendix 1).

While it has been reported that PWS are prone to relapse in the absence of booster therapy, the participant’s scores continued to improve across the four testing phases, with continued self-improvement in the 24 months before the final assessment, without any additional support events. In addition, while secondary behaviours were not specifically targeted, there was a reduction in physical contaminants, duration of dysfluency and increased speech naturalness across the four testing periods. This again implies unconscious tongue positioning plays a role in the subsequent chain of stuttering events for PWS.

### Objective 2

#### Activity limitations, participation restrictions and contextual factors

By 24 months follow-up, the negative impact of being someone who stutters, as assessed with OASES-A, had been reduced from moderately severe to mild–moderate, with a 29% total reduction in scaled score from baseline. This scale is seen as a good indicator of therapy outcomes, more so than stuttering severity, and is less subject to variability (Yaruss, 2010). Therefore, any improvements in this section represent real change. The sub-section scores for Quality of Life showed the same improvement as the overall impact score: from moderate–severe to mild–moderate reflected in a 37% reduction in negative impact score from baseline.

General Information score was reduced initially from moderate–severe to moderate, but this had reverted to moderate–severe at Phase 5. Speaker’s Reactions score reduced by 39% initially to mild but stabilised at mild–moderate (19% improvement) indicating they did not have as many negative reactions as before the intervention. Daily Communication improved from moderate to mild–moderate, although this decreased across phases 3–5 (from 20% to 10% improvement).

These results are comparable to those reported following a comprehensive stuttering intervention (Karani & Mupawose, 2020) where individual 1-h sessions were given once a week for 7 months (Total OASES-A score 34% reduction from baseline); 12-week Communication Effectiveness Intervention (average Total OASES-A 23% reduction from baseline) (Byrd et al., 2022); and MIST delivered in 10 h over four sessions (average Total OASES-A score 10% reduction from baseline) (Sønsterud et al., 2020). This intervention was delivered online in less than 2 h spread over 5 weekly sessions and the total OASES-A score reduced by 29%. The greatest improvement was in Quality of Life, and as this is a core component within ICF classification, it is a very promising result.

### Objective 3

#### Participants perspective – Self report

Further analysis of ICF components is provided through the Clinical Use Self-Report (CUSR) within SSI-4. The participant self-reported that their average stuttering severity had improved with a reduction of 19% from the baseline score, his frequency of avoidance behaviours decreased initially by 19% and then further improved to 24% (phases 4 and 5), and he had an overall increased internal locus of control with a 31% change in score from baseline. In line with the ICF components these measures reflect improved function (19% change from baseline) reduced activity limitations and participation restrictions (24% change from baseline) and improved personal and environmental factors (31% change from baseline).

The CUSR has a high test–retest reliability, and there was no significant correlation between any of the three CUSR items and the three main parameters within the SSI-4 (Tahmasebi et al., 2018). As such, these items complement the main SSI-4, and while it was only developed for clinical use, it provides important research information about these parameters. Self-rated stuttering severity at Phase 5 (4.1) corresponds to the equivalent score (4.6) in the SRSS tool added at Phase 5. This supports the validity of the measures used.

The unpredictable and variable nature of a PWS’ ability to achieve speech motor control has been highlighted as a compounding factor in subsequent negative reactions for PWS (Tichenor et al., 2022). Using a conscious eye movement, the participant’s locus of control score decreased which suggests an increased internal locus of control or ability to consciously decide whether to stutter or not. This outcome suggests that unconscious predictions reflected in unconscious eye movement play a significant role in the aetiology of stuttering.

### Objective 4

#### Premonitory awareness – Immediate and prospective anticipation

The PAiS score reduced as the severity was reduced. Increases in PAiS have previously correlated with reduced %SS (Cholin et al., 2016) when the tool was psychometrically validated. This had been interpreted as a skilled application of standard fluency techniques. This intervention produced the opposite effect, with a reduction in premonitory sensations stabilising at 56% at 24 months follow-up assessment (Phase 5). This suggests that this is not like any other fluency-enabling tool, and there is a distinct qualitative difference in what the participant was doing.

Cholin et al. (2016) criticised the PAiS scale for not differentiating between immediate and prospective awareness; however, the this study considers that as 6 of 12 items commenced with ‘right before I stutter…’, these specifically dealt with immediate anticipation and the reduction in these appears to account for most variation observed. This suggests that a targeted eye movement may directly address the cause of anticipatory sensations and the stuttering-like moments they predict. By avoiding the stuttering event, subsequent negative outcomes from the predicted chain of events do not occur. By avoiding the stuttering event, subsequent negative outcomes from the predicted chain of events do not occur.

At 24 months follow-up, Q14 scores were not included in the results as the question was found to reduce the internal consistency of the PAiS tool when it was formally validated (Cholin et al., 2016). The final question asked the participant to rate the statement ‘I really mind that I stutter’. This improved from being very much true (3) to a little true (1) at Phase 5 (full results are available from the corresponding author on request).

### Objective 5

#### Self-report stuttering severity

By converting the SRSS scores to comparative %SS based on the published comparison table (Jones et al., 2005), these subjective scores can be compared to the objective %SS at the final assessment via SSI-4. The participant’s actual percentage of stuttered syllables was greater (16.55%) than he estimated it to be (12.6% approximately) through self-assessment when speaking. When reading, the objectively assessed performance was significantly better (0.53%) than the participant’s self-assessment (2.6% approximately). This represents an average score of 8.54%, corresponding well with the SRSS typical severity (8.6% approximately). This supports the use of average score SRSS when expanding the research.

The participant also reported on avoidance behaviours. This was assessed in this tool on a scale of 1 (rarely) to 3 (frequently). The participant said he rarely avoided seven out of the eight speaking situations. This correlates to the reduction of frequency of avoidance behaviours assessed through the SSI-4 CUSR and supports the validity of the measures used in this report.

The participant rated their satisfaction with speech as 2, with 1 being extremely satisfied and 9 extremely dissatisfied. This was at Phase 5. The participant’s concluding comments regarding the technique, 24 months after participating were ‘Found it very useful. Is difficult to remember to do it but when I remember it really helps’. The total participant contact time was less than 2 h over a 5-week period online and 5 weeks of daily text messages. This is especially relevant as, as far as challenges to practice are concerned, COVID-19 has significantly increased the workload of clinicians (Khoza-Shangase & Mophosho, 2021).

## Conclusion

This descriptive case report used a variety of tools to assess the effectiveness and feasibility of this novel therapeutic approach. The participant’s scores improved on all independent and self-assessed measures used in this exploration. This may reflect an improvement in all components of stuttering: core behaviour, secondary behaviour and negative affect. It also reflects the participant successfully using the technique in real life post-intervention. Unlike restructuring interventions, the participant’s speech naturalness improved, and his experience of premonitory sensations was reduced across testing phases. For this participant making a specific timed conscious eye movement was a feasible intervention producing holistic clinical benefits.

This approach represents a qualitative change in speech, language and hearing research and the start of an evidence base for a service delivery model that may be culturally, linguistically and contextually compatible with the diverse South African population. There will always be a need for individualised, comprehensive treatments, and with further research, these could include this tool to improve patient outcomes. For those who cannot access mainstream service delivery, this technique on its own may produce clinically significant results, as it did for this participant.

The intervention was delivered remotely with minimum contact time and support. As the instructions concern a physical movement, understanding and following translation would be easy to establish. This intervention, with further research, could provide a pathway to improve the Quality of Life of the most vulnerable in the South African and Worldwide stuttering community. The results support further exploration at the pilot randomised control trial (NCT04310436), so that causality can be determined, and the results are more generalisable to the stuttering community. The link between non-vision-related eye movement and tongue position needs more scientific exploration as conscious eye movement may be a valuable clinical tool for stuttering.

## Appendix 1

**TABLE 1-A1:** Stuttering Severity Instrument 4 results.

Description	Reading frequency (SSI)			Speaking frequency (SSI)			Combined frequency 1 decimal place		Frequency/duration of longest stuttered syllables (SSI)			Physical concomitants (SSI)/naturalness of speech		Total SSI score and category	Percentile
	SSI-4 raw scores	Scaled scores	% decrease	SSI-4 raw scores	Scaled scores	% decrease	SSI-4 raw scores	% decrease	SSI-4 raw scores	Scaled scores	% decrease	Scaled scores	% decrease		
Phase 1 Baseline	17.3	8	-	30.8	9	-	48.1	-	17/1.56s	6	-	(6)/7	-	29§	61–67 41–77
Phase 3 % reduction from baseline	13.7	8	21	23.48	9	31	37.2	23	17/1.45ss	6	7	(2)/3†	66	25§	41–60 41–77
Phase 4 % reduction from baseline	8.35	7	51.8	23.7	9	23	32	34	15/0.9s	4	43	(3)/4.5‡	50	23¶	24–40
Phase 5 % reduction from baseline	0.53	2	97	16.55	8	46	17.1	65	10/0.48s	2	70	(3)/4.5‡	50	15††	4–11

† , 66% of the scaled score;

‡ , 50% of the scaled score;

§ , moderate;

¶ , mild;

†† , very mild.

## Appendix 2

### Overall assessment of the speaker’s experience of stuttering - Adults.

**Table:** Section 1: General information

A: General information	17	10	14	13
B: Knowledge	12	9	9	15
C: Feelings	35	24	26	34
Section total/*n* = number answered	64/20	43/20	49/20	62/20
Section impact score	3.2	2.15	2.45	3.1
Section impact rating	Moderate severe	Moderate	Moderate	Moderate severe
% reduction from the baseline section total score	-	32.8	23.5	2

**Table:** Section 2: Your reactions to stuttering

A: Affective	34	18	29	29
B: Behavioural	34	17	23	26
C: Cognitive	33	26	30	28
Section total/*n* = number answered	101/30	61/30	82/30	83/30
Section impact score	3.37	2.03	2.8	2.8
Section impact rating	Moderate-severe	Mild-moderate	Moderate	Moderate
% reduction from the baseline section total score	-	39	19	19

**Table:** Section 3: Communication in daily situations

A: Social situations	11	11	10	10
B: At home	8	4	7	7
Section total/*n* = number answered	54/23	45/24	45/23	49/23
Section impact score	2.35	1.88	1.96	2.13
Section impact rating	Moderate	Mild-moderate	Mild-moderate	Mild-moderate
% reduction from the baseline section total score	-	20	16.6	9.4

**Table:** Section 4: Quality of life

A: Quality of life	9	6	6	6
B: Satisfaction with communication	12	7	8	8
C: Interpersonal	15	7	9	7
D: Social	17	10	13	10
E: Intrapersonal	24	16	25	18
Section total/*n* = number answered	77/25	46/25	61/25	49/25
Section impact score	3.08	1.84	2.44	1.96
Section impact rating	Mod-severe	Mild-moderate	Moderate	Mild-moderate
% reduction from the baseline section total score	-	40	21	37

**Table:** Overall impact

Total/*n* = number answered	402/98	195/99	237/98	255/98
Impact score	3.02	1.97	2.42	2.16
Impact rating	Mod-severe	Mild-moderate	Moderate	Mild-moderate
% reduction from the baseline section total score	-	35	20	29

## References

[CIT0001] American Speech-Language-Hearing Association (ASHA). (2016). Scope of practice in Speech Language Pathology [Scope of Practice]. Available from https://www.asha.org/policy/SP2016-00343/

[CIT0002] Barratt, J., Khoza-Shangase, K., & Msimang, K. (2012). Speech-language assessment in a linguistically diverse setting: Preliminary exploration of the possible impact of informal ‘solutions’ within the South African context. *South African Journal of Communication Disorders*, 59(1), a20. 10.4102/sajcd.v59i1.20 23409617

[CIT0003] Blumgart, E., Tran, Y., & Craig, A. (2010). An investigation into the personal financial costs associated with stuttering. *Journal of Fluency Disorders*, 35(3), 203–215. 10.1016/j.jfludis.2010.03.002 20831968

[CIT0004] Boyle, M.P., & Chagachbanian, N.J. (2022). Uncertainty and perceived control as predictors of communicative participation and mental health in adults who stutter. *American Journal of Speech-Language Pathology*, 31(2), 757–769. 10.1044/2021_AJSLP-21-00230 35007427

[CIT0005] Brignell, A., Krahe, M., Downes, M., Kefalianos, E., Reilly, S., & Morgan, A.T. (2020). A systematic review of interventions for adults who stutter. *Journal of Fluency Disorders*, 64, 105766. 10.1016/j.jfludis.2020.105766 32438123

[CIT0006] Briley, P.M. (2016). An exploration of anticipation of stuttering in adults. *Journal of Speech Pathology & Therapy*, 02(01). 10.4172/2472-5005.1000123

[CIT0007] Byrd, C.T., Coalson, G.A., & Young, M.M. (2022). Targeting communication effectiveness in adults who stutter: A preliminary study. *Topics in Language Disorders*, 42(1), 76–93. 10.1097/TLD.0000000000000270

[CIT0008] Carey, B., O’Brian, S., Onslow, M., Block, S., Jones, M., & Packman, A. (2010). Randomized controlled non-inferiority trial of a telehealth treatment for chronic stuttering: The camperdown program. *International Journal of Language and Communication Disorders*, 45(1), 108–120. 10.3109/13682820902763944 19424889

[CIT0009] Carey, B., O’Brian, S., Onslow, M., Packman, A., & Menzies, R. (2012). Webcam delivery of the camperdown program for adolescents who stutter: A phase I trial. *Language, Speech, and Hearing Services in Schools*, 43(3), 370–380. 10.1044/0161-1461(2011/11-0010) 22232423

[CIT0010] Cholin, J., Heiler, S., Whillier, A., & Sommer, M. (2016). Premonitory Awareness in Stuttering Scale (PAiS). *Journal of Fluency Disorders*, 49, 40–50. 10.1016/j.jfludis.2016.07.001 27638191

[CIT0011] Connery, A., Galvin, R., & McCurtin, A. (2021). Effectiveness of nonpharmacological stuttering interventions on communication and psychosocial functioning in adults: A systematic review and meta-analysis of randomized controlled trials. *Journal of Evidence-Based Medicine*, 14(1), 17–26. 10.1111/jebm.12408 33242235

[CIT0012] Connery, A., Yaruss, J.S., Lomheim, H., Loucks, T.M., Galvin, R., & McCurtin, A. (2022). Obtaining consensus on core components of stuttering intervention for adults: An e-Delphi Survey with key stakeholders. *International Journal of Language and Communication Disorders*, 57(1), 112–127. 10.1111/1460-6984.12680 34818457

[CIT0013] Constantino, C.D., Eichorn, N., Buder, E.H., Gayle Beck, J., & Manning, W.H. (2020). The speaker’s experience of stuttering: Measuring spontaneity. *Journal of Speech, Language, and Hearing Research*, 63(4), 983–1001. 10.1044/2019_JSLHR-19-00068 32213101

[CIT0014] Craig, A., Hancock, K., Tran, Y., Craig, M., & Peters, K. (2002). Epidemiology of stuttering in the community Across the Entire Life Span. *Journal of Speech, Language, and Hearing Research*, 45(6), 1097–1105. 10.1044/1092-4388(2002/088) 12546480

[CIT0015] Di Vico, R., Paolo Ardigò, L., Salernitano, G., Chamari, K., & Padulo, J. (2013). The acute effect of the tongue position in the mouth on knee isokinetic test performance: A highly surprising pilot study. *Ligaments and Tendons Journal*, 3(4), 318–323. PMC3940506 24596696

[CIT0016] Diaz, G., Cooper, J., Rothkopf, C., & Hayhoe, M. (2012). Internal models for predictive Saccades in a natural interception task. *Journal of Vision*, 12(9), 606. 10.1167/12.9.606

[CIT0017] Didirkova, I., Le Maguer, S., Hirsch, F., Gbedahou, D., Articulatory, D.G., & Didirková, I. (2019). *Articulatory behaviour during disfluencies in stuttered speech*, Retrieved from https://halshs.archives-ouvertes.fr/halshs-02281284

[CIT0018] Guitar, B. (2014). *Stuttering: An integrated approach to its nature and treatment*, Lippincott Williams & Wilkins.

[CIT0019] Jackson, E.S., Yaruss, J.S., Quesal, R.W., Terranova, V., & Whalen, D.H. (2015). Responses of adults who stutter to the anticipation of stuttering. *Journal of Fluency Disorders*, 45, 38–51. 10.1016/j.jfludis.2015.05.002 26065618 PMC4728710

[CIT0020] Jones, M., Onslow, M., Packman, A., Williams, S., Ormond, T., Schwarz, I., & Gebski, V. (2005). Randomised controlled trial of the Lidcombe programme of early stuttering intervention. *British Medical Journal*, 331(7518), 659–661. 10.1136/bmj.38520.451840.E0 16096286 PMC1226241

[CIT0021] Karani, T.F., & Mupawose, A. (2020a). A descriptive analysis of assessment measures on the effectiveness of a comprehensive stuttering intervention approach: A single case study. *South African Journal of Communication Disorders*, 67(1), 1–9. 10.4102/SAJCD.V67I1.648 PMC7203267 32370524

[CIT0022] Karrim, S.B., Flack, P.S., Naidoo, U., Beagle, S., & Pontin, A. (2022). The experiences of speech-language therapists providing telerehabilitation services to children with autism spectrum disorder. *South African Journal of Communication Disorders*, 69(2). 10.4102/sajcd.v69i2.917 PMC9453137 36073081

[CIT0023] Khoza-Shangase, K., & Mophosho, M. (2018). Language and culture in speech-language and hearing professions in South Africa: The dangers of a single story. *South African Journal of Communication Disorders*, 65(1), a594. 10.4102/sajcd.v65i1.594 PMC6111603 30035607

[CIT0024] Khoza-Shangase, K., & Mophosho, M. (2021). Language and culture in speech-language and hearing professions in South Africa: Re-imagining practice. *South African Journal of Communication Disorders*, 68(1). 10.4102/sajcd.v68i1.793 PMC8252163 34082547

[CIT0025] Kosmala, L., Candea, M., & Morgenstern, A. (2019). Synchronization of (dis)fluent speech and gesture: A multimodal approach to (dis)fluency. In Paderborn (Ed.), *Gesture and Speech in Interaction* (6th edn.) (pp. 56–61). Paderborn, Germany. hal-02360613

[CIT0026] Langevin, M., Kully, D., Teshima, S., Hagler, P., & Narasimha Prasad, N. G. (2010). Five-year longitudinal treatment outcomes of the ISTAR Comprehensive Stuttering Program. *Journal of Fluency Disorders*, 35(2), 123–140. 10.1016/j.jfludis.2010.04.002 20609333

[CIT0027] Lohr, D., & Komogortsev, O.V. (2022). Eye know you too: Toward viable end-to-end eye movement biometrics for user authentication. *IEEE Transactions on Information Forensics and Security*, 17, 3151–3164. 10.1109/TIFS.2022.3201369

[CIT0028] Maguire, G., Franklin, D., Vatakis, N.G., Morgenshtern, E., Denko, T., Yaruss, J.S., Spotts, C., Davis, L., Davis, A., Fox, P., Soni, P., Blomgren, M., Silverman, A., & Riley, G. (2010). Exploratory randomized clinical study of pagoclone in persistent developmental stuttering: The examining pagoclone for persistent developmental stuttering study. *Journal of Clinical Psychopharmacology*, 30(1), 48–56. 10.1097/JCP.0b013e3181caebbe 20075648

[CIT0029] McDonagh, H., & Monaghan, K. (2019). Neuroplastic control of developmental stutter via the frontal Aslant tract: Past, present, and future. *American Journal of Biomedical Science & Research*, 5(6), 507–511. 10.34297/ajbsr.2019.05.000978

[CIT0030] Meredith, G., Achterbosch, L., Peck, B., Terry, D., Dekker, E., & Packman, A. (2023). The use of an interactive social simulation tool for adults who stutter: A pilot study. *European Journal of Investigation in Health, Psychology and Education*, 13(1), 187–198. 10.3390/ejihpe13010014 36661764 PMC9858588

[CIT0031] Murad, H., Asi, N., Alsawas, M., & Alahdab, F. (2016). New evidence pyramid, *Evidence-Based Medicine*, 21(4), 125–127. 10.1136/ebmed 27339128 PMC4975798

[CIT0032] O’Brian, S., Packman, A., Onslow, M., & O’Brian, N. (2004). Measurement of stuttering in adults. *Journal of Speech Language and Hearing Research*, 47(5), 1081. 10.1044/1092-4388(2004/080) 15603463

[CIT0033] Reddy, R.P., Sharma, M.P., & N.S. (2010). Cognitive behavior therapy for stuttering: A case series. *Indian Journal of Psychological Medicine*, 32(1), 49–53. 10.4103/0253-7176.70533 21799560 PMC3137813

[CIT0034] Riley, G.D. (1994). Stuttering severity instrument for children and adults (SSI-3) 3rd ed. Pro Ed; Austin: TX. 10.1044/jshd.3703.314 5057250

[CIT0035] Riley, G.D. (2009). Stuttering severity instrument for children and adults (SSI-4) 4th ed. Pro-Ed, Inc; Austin: TX. 10.1044/jshd.3703.314 5057250

[CIT0036] Reilly, S., Onslow, M., Packman, A., Wake, M., Bavin, E.L., Prior, M., Eadie, P., Cini, E., Bolzonello, C., & Ukoumunne, O.C. (2009). Predicting stuttering onset by the age of 3 years: A prospective, community cohort study. *Pediatrics*, 123(1), 270–277. 10.1542/peds.2007-3219 19117892 PMC3879585

[CIT0037] Riva-Posse, P., Busto-Marolt, L., Schteinschnaider, Á., Martinez-Echenique, L., Cammarota, Á., & Merello, M. (2008). Phenomenology of abnormal movements in stuttering. *Parkinsonism and Related Disorders*, 14(5), 415–419. 10.1016/j.parkreldis.2007.11.006 18316236

[CIT0038] Schmidt, J.E., Carlson, C.R., Usery, A.R., & Quevedo, A.S. (2009). Effects of tongue position on mandibular muscle activity and heart rate function. *Oral Surgery, Oral Medicine, Oral Pathology, Oral Radiology and Endodontology*, 108(6), 881–888. 10.1016/j.tripleo.2009.06.029 19773187

[CIT0039] Sønsterud, H., Halvorsen, M.S., Feragen, K.B., Kirmess, M., & Ward, D. (2020). What works for whom? Multidimensional individualized stuttering therapy (MIST). *Journal of Communication Disorders*, 88, 106052. 10.1016/j.jcomdis.2020.106052 33080388

[CIT0040] Tahmasebi, N., Shafie, B., Karimi, H., & Mazaheri, M. (2018). A Persian-version of the stuttering severity instrument-version four (SSI-4): How the new additions to SSI-4 complement its stuttering severity score? *Journal of Communication Disorders*, 74, 1–9. 10.1016/j.jcomdis.2018.04.005 29723653

[CIT0041] Tar-Mahomed, Z., & Kater, K.A. (2022). The perspectives of speech–language pathologists: Providing teletherapy to patients with speech, language and swallowing difficulties during a COVID-19 context. *South African Journal of Communication Disorders*, 69(2), a902. 10.4102/sajcd.v69i2.902 PMC9452921 36073074

[CIT0042] Tenison, C., Fincham, J.M., & Anderson, J.R. (2016). Phases of learning: How skill acquisition impacts cognitive processing. *Cognitive Psychology*, 87, 1–28. 10.1016/J.COGPSYCH.2016.03.001 27018936

[CIT0043] Tichenor, S.E., Herring, C., & Yaruss, J.S. (2022). Understanding the speaker’s experience of stuttering can improve stuttering therapy. *Topics in Language Disorders*, 42(1), 57–75. 10.1097/TLD.0000000000000272 35757374 PMC9231935

[CIT0044] Woods, D.W., Piacentini, J., Himle, M.B., & Chang, S. (2005). Premonitory Urge for Tics Scale (PUTS): Initial psychometric results and examination of the premonitory urge phenomenon in youths with tic disorders. *Developmental and Behavioural Pediatrics*, 26(6), 397–403. 10.1097/00004703-200512000-00001 16344654

[CIT0045] World Health Organization (WHO). (2001). International Classification of Functioning, Disability, and Health, International Classification of Functioning, Disability and Health. Geneva: ICF. Available at: https://apps.who.int/iris/handle/10665/42407

[CIT0046] World Health Organization (WHO). (2016). *International statistical classification of diseases and related health problems, 10th Revision ICD-10* (10th edn.).

[CIT0047] Yairi, E., & Ambrose, N. (2013). Epidemiology of stuttering: 21st century advances. *Journal of Fluency Disorders*, 38(2), 66–87. 10.1016/j.jfludis.2012.11.002 23773662 PMC3687212

[CIT0048] Yaruss, J.S., & Quesal, R.W. (2004). Stuttering and the International Classification of Functioning, Disability, and Health (ICF): An update. *Journal of Communication Disorders*, 37(1), 35–52. 10.1016/S0021-9924(03)00052-2 15013378

[CIT0049] Yaruss, J.S. (2010). Assessing quality of life in stuttering treatment outcomes research. *Journal of Fluency Disorders*, 35(3), 190–202. 10.1016/J.JFLUDIS.2010.05.010 20831967

[CIT0050] Yaruss, J., Coleman, C.E., & Quesal, R.W. (2012). Stuttering in school-age children: A comprehensive approach to treatment. *Language, speech, and hearing services in schools*, 43(4), 536–548. 10.1044/0161-1461(2012/11-0044) 23047437

